# LINC00472 inhibits cell migration by enhancing intercellular adhesion and regulates H3K27ac level via interacting with P300 in renal clear cell carcinoma

**DOI:** 10.1038/s41420-022-01243-7

**Published:** 2022-11-12

**Authors:** Songmao Wang, Cheng Luo, Bing Li, Shikuan Zhang, Weijie Liao, Qilei Xin, Naihan Xu, Weidong Xie, Yuanchang Zhu, Yaou Zhang

**Affiliations:** 1 School of Life Sciences, Tsinghua University, Beijing, 100084 China; 2 China State Key Laboratory of Chemical Oncogenomics, Tsinghua Shenzhen International Graduate School, Shenzhen, 518055 China; 3 Key Lab in Healthy Science and Technology of Shenzhen, Tsinghua Shenzhen International Graduate School, Shenzhen, 518055 China; 4 Department of Biomedical Engineering, Tsinghua University, Beijing, 100084 China; 5 Open FIESTA Center, Tsinghua University, Shenzhen, 518055 China; 6 Reproductive Medicine Center, Shenzhen Hengsheng Hospital, Shenzhen, 518115 China

**Keywords:** Extracellular matrix, Long non-coding RNAs

## Abstract

Renal clear cell carcinoma (RCCC) is the most common type of renal cell carcinoma, which is also difficult to diagnose and easy to metastasize. Currently, there is still a lack of effective clinical diagnostic indicators and treatment targets. This study aims to find effective diagnostic markers and therapeutic targets from the perspective of noncoding RNA. In this study, we found that the expression of Long noncoding RNA LINC00472 was significantly decreased in RCCC and showed a downward trend with the progression of cancer stage. Patients with low LINC00472 expression have poor prognosis. Inhibition of LINC00472 significantly increased cell proliferation and migration, while overexpression of LINC00472 obviously inhibited cell proliferation and enhanced intercellular adhesion. Transcriptome sequencing analysis demonstrated that LINC00472 was highly correlated with extracellular matrix and cell metastasis-related pathways, and the consistent results were obtained by The Cancer Genome Atlas (TCGA) data analysis. Additionally, we discovered that the integrin family protein ITGB8 is a potential target gene of LINC00472. Mechanistically, we found that the change of LINC00472 affected the acetylation level of H3K27 site in cells, and we speculate that this effect is likely to be generated through the interaction with acetyltransferase P300. In conclusion, LINC00472 has an important impact on the proliferation and metastasis of renal clear cells, and probably participate in the regulation of histone modification, and it may be used as a potential diagnostic marker of RCCC.

## Introduction

Renal cell carcinoma (RCC) is the most common and fatal cancer in the urinary system. Among all RCC types, renal clear cell carcinoma (RCCC) accounts for about 80% and it is also the most aggressive renal tumor [1, 2]. The etiology of RCCC is complex, the related factors include genetic factors (such as deletion or inactivation of VHL gene), obesity, hypertension, or antihypertensive treatment [3, 4]. At present, the main obstacle of RCCC treatment is the lack of diagnostic markers and effective specific therapeutic targets. Patients with RCCC often have developed to advanced stage when diagnosed, and most of them have metastasized [5, 6]. Therefore, finding reliable diagnostic factors and effective therapeutic targets is important to improve the therapeutic effect of RCCC.

Long noncoding RNAs (lncRNAs), which are functionally defined as transcripts >200 nt in length with no protein-coding potential, have been found to be involved in a variety of cellular functions [7–9]. The main modes of action of lncRNA include: participating in the regulation of histone modification to change the conformation of chromatin and regulate gene transcription [10, 11], acting as scaffolds to promote the binding of protein to protein (or DNA), or directly interact with proteins to affect their localization or activity [12], and playing as a “sponge”, competitively bind to miRNAs and then regulate the expression of target genes [13–15]. It is worth noting that lncRNAs play important roles in multiple cancers, and the functions have been summarized in six aspects: proliferation, growth suppression, motility, immortality, angiogenesis, and viability [16–18]. Significantly, compared with the genes encoding proteins, most lncRNAs are non-conservative and tissue-specific, which provide a better choice for finding new disease treatment targets [19–21].

In this study, we found that the expression of lncRNA LINC00472 was significantly reduced in RCCC, and the prognosis of patients with low expression LINC00472 were poor. Moreover, we verified LINC00472 biological functions and found that it can inhibit cell proliferation and migration. Transcriptome sequencing analysis and TCGA data analysis indicated that LINC00472 was highly correlated with extracellular matrix and cell metastasis-related pathways. Mechanistically, our results found that LINC00472 may through interact with acetyltransferase P300 to regulate the level of H3K27ac in cells.

## Results

### Construction of WGCNA co-expression module

**Fig. 1:**
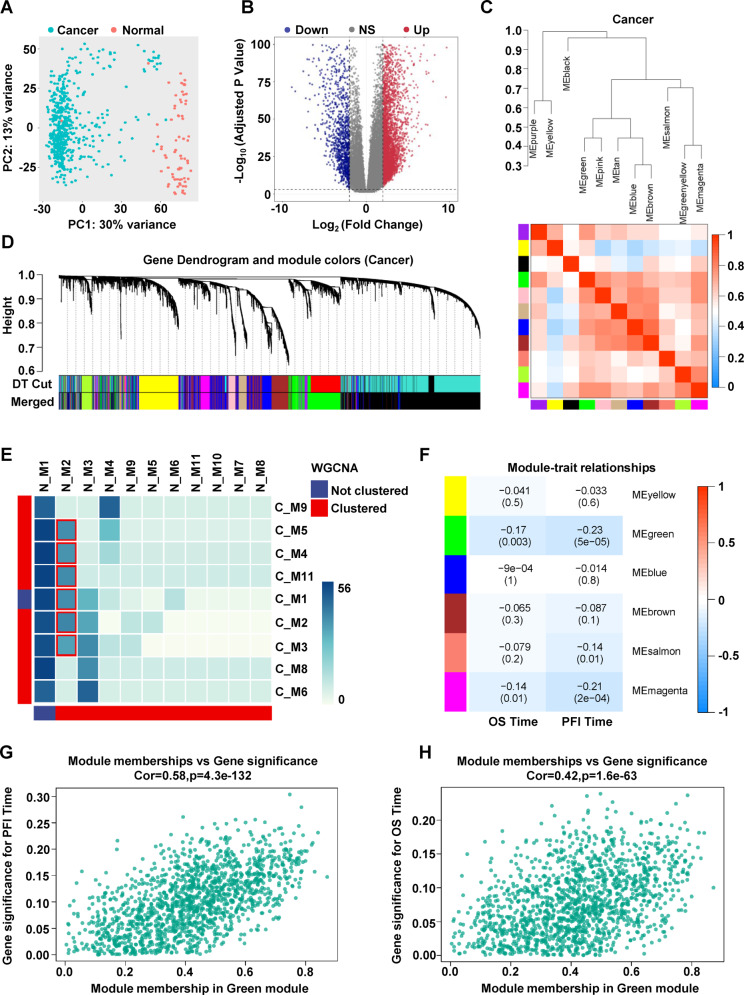
Weighted gene co-expression network analysis. **A** Principal component analysis of RCCC cancer group and normal groups. **B** Volcano plot of differentially expressed genes between cancer and normal groups. **C** According to the combination of module dissimilarity, 11 sub modules were finally obtained in the cancer group. **D** The correlation between cancer network modules. **E** Cross analysis of cancer module and normal module, color block represents the number of overlapping genes. **F** Heat map showing correlation between the gene module and clinical traits. **G** The relevance of module membership in green and gene significance for PFI time. **H** The relevance of module membership in green and gene significance for OS time.

We divided RCCC samples into cancer and normal group according to the notes of TCGA and the Principal Component Analysis (PCA) showed that the samples could be divided into two obvious clusters (Fig. 1A). Then, we analyzed differentially expressed genes in both groups and identified 6303 upregulated and 3563 downregulated genes (|Log_2_ Fold Change| > 1, *p*-value < 0.001). The Log_2_ of enrichment ratio and −Log_10_ of adjusted p-value were visualized in the volcano plot (Fig. 1B). Based on fold change, we selected 20000 genes with the most obvious absolute value change among the differentially expressed genes and performed weighted gene co-expression network analysis (WGCNA) in cancer and normal samples. According to the average connectivity of the network, we selected the appropriate soft threshold (beta) in cancer samples and normal samples respectively (Fig. S1A, C), and detected the node connection number of scale-free network under the selected soft threshold (Fig. S1B, D). The fractional-step algorithm is used to construct modules and merged sub modules with dissimilarity less than 0.3 (Figs. 1C and S2A), and 11 co-expression networks were finally obtained in cancer and normal group respectively (Figs. 1D and S2B).

We analyzed WGCNA results of two groups and found that there were some overlapping genes in different types of data modules. In order to further excavate meaningful genes, we performed cross analysis on the two groups data. Excluding C_Mod1 and N_Mod1 that cannot be clustered, we found that N_Mod2 and several cancer modules (C_M5, C_M4, C_M11, C_M1, C_M1, C_M2, C_M3) intersect significantly, suggesting that the expression pattern of these overlapping genes in cancer may have changed (Fig. 1E). Next, we analyzed the correlation between these six cancer modules and patient clinical survival time. The correlation coefficient in each pattern represented the relation between gene module and the clinical traits, which decreased in size from red to blue. Most notably, we found that there was a significant correlation between the green module (C_Mod4) and the patient clinical prognosis survival time (Fig. 1F). Next, we analyzed the correlation between green module genes and patient survival (PFI time and OS time), and the results showed most genes in this module have a strong correlation with patient survival (Fig. 1G, H).

### Renal clear cell carcinoma patients with low expression of LINC00472 have poor prognosis

**Fig. 2:**
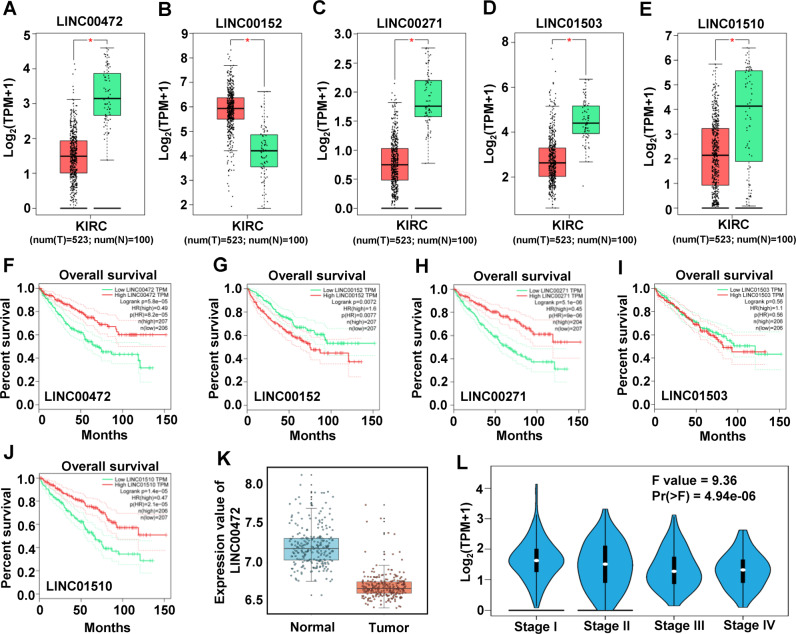
LINC00472 is low expressed in RCCC and is related to the prognosis of patients. Expression of LINC00472 (**A**), LINC00152 (**B**), LINC00271 (**C**), LINC01503 (**D**), and LINC01510 (**E**) of TCGA RCCC samples, red represents tumor group and green represents normal group. **F**–**J** Relationship between the levels of expression of LINC00472 (**F**), LINC00152 (**G**), LINC00271 (**H**), LINC01503 (**I**), and LINC01510 (**J**) and the prognosis of patients (OS time). **K** Expression of LINC00472 in GSE44055 datasets. **L** Expression of LINC00472 in TCGA RCCC samples with different tumor stage.

After determining the module which most closely related to patient survival, we analyzed the expression of lncRNAs in this module, and finally obtained five lncRNAs (LINC00472, LINC00152, LINC00271, LINC01503, LINC01510) with significant differences in expression (Fig. 2A–E). Next, we analyzed the relationship between the expression of these lncRNAs and patient survival. As shown in Figs. 2F–J and S3A–E, LINC00472, LINC00152, LINC00271 and LINC01510 had a strong relationship with the survival time of patients. LINC00271 is highly expressed in testis and has a relative low expression in kidney, while LINC00472 and LINC01510 are highly expressed in renal tissue. Previous studies have shown that LINC01510 and LINC00152 play important roles in RCCC, which indicating that our bioinformatics analysis is reliable and effective. But it is still not clear what function LINC00472 plays in RCCC, so we next carried out the study on the function and mechanism of LINC00472. We selected a group of samples from RCCC and adjacent tissues in GEO database (GSE40435), and analyzed the expression of LINC00472. The results also showed that LINC00472 decreased significantly in cancer tissues comparing with the adjacent tissues (Fig. 2K). In addition, we found that the level of LINC00472 decreased gradually with the progress of cancer stage (Fig. 2L). According to the results of bioinformatics analysis, we speculated that the deletion of LINC00472 may promote the development of RCCC.

### Knockdown of LINC00472 expression increased cell proliferation and migration

**Fig. 3:**
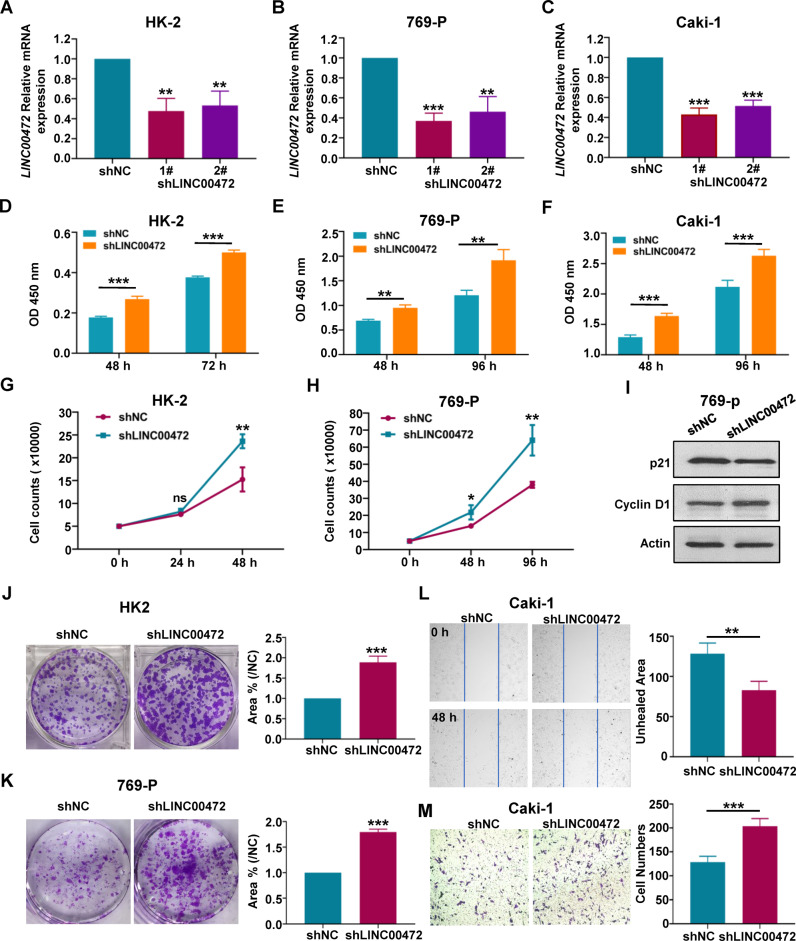
Inhibition of LINC00472 promotes cell proliferation and migration. HK-2 (**A**), 769-P (**B**), and Caki-1 (**C**) cells were transfected with control shRNA vectors (shNC) and the Lentivirus-based LINC00472-targeting shRNA (shLINC00472-1# and shLINC00472-2#), and the inhibition efficiency of lentivirus interference plasmid was detected by RT-qPCR. Cell Counting Kit-8 test was used to detect the proliferation of HK-2 (**D**), 769-P (**E**), and Caki-1 (**F**) cell lines. **G**, **H** Cell counts was used to compare the proliferation rate of LINC00472 stable knockdown cell line and control cell line of HK-2 (**G**) and 769-P cells (**H**). **I** The expression of cell proliferation-related proteins in 769-P shNC and 769-P shLINC00472 cell lines was detected by using western blot. **J** Colony formation assay of HK-2-shLINC00472 cell line and HK-2-shNC cell line (Left). Statistics of clone formation Area (Right). **K** Colony formation assay of 769-P shLINC00472 cell line and 769-P shNC cell line (Left). Statistics of clone formation Area (Right). **L** Wound healing experiment was used to detect the migration of Caki-1 shLINC00472 cell line and the control cell line. **M** Using transwell to detect the migration of Caki-1 shNC and shLINC00472 cell line (Left). Statistics of migrating cell numbers (Right). In **A**–**M**, data were shown as mean ± SD of three independent experiments (*P < 0.05, **P < 0.01, ***P < 0.001, ns non-significant).

We transfected HK-2, 769-P, and Caki-1 cells with LINC00472 lentivirus interference plasmid and control plasmid respectively, and using RT-qPCR to detect the mRNA expression of LINC00472. The results showed that the expression of LINC00472 in cells transfected with lentivirus interference plasmid was significantly decreased (Fig. 3A–C). After puromycin screening, we obtained the HK-2 shLINC00472, 769-P shLINC00472, and Caki-1 shLINC00472 cell lines with LINC00472 stable low expression, as well as their corresponding control cell lines.

Subsequently, we detected the cell proliferation ability by using cell counting kit-8 (CCK-8) and cell count. The CCK-8 results indicated that inhibition of LINC00472 significantly increased the cell proliferation (Fig. 3D–F). The results of cell count were consistent with CCK-8 (Figs. 3G, H and S4A). Moreover, we checked the level of proteins related to cell proliferation by using western blot, and the results showed that the levels of p21 and p27 protein which inhibited cell proliferation significantly decreased in LINC00472 low expression cell lines comparing with control cell line, while the level of CyclinD1, CyclinB1 and CDK4 protein which promoted cell proliferation was significantly increased (Figs. 3I and S4B). Single cell clone formation experiment showed that inhibition of LINC00472 obviously enhanced the ability of cell clone formation (Figs. 3J, K and S4C). Furthermore, wound healing experiment and transwell experiment demonstrated that knockdown of LINC00472 significantly increased cell migration in Caki-1 cell (Fig. 3L, M) and 769-P cell (Fig. S4D, E). The above experimental results showed that inhibition of LINC00472 promotes cell proliferation and migration in RCCC cell.

### Construction of LINC00472 knockdown and overexpression stable cell line by CRISPR-Cas9

**Fig. 4:**
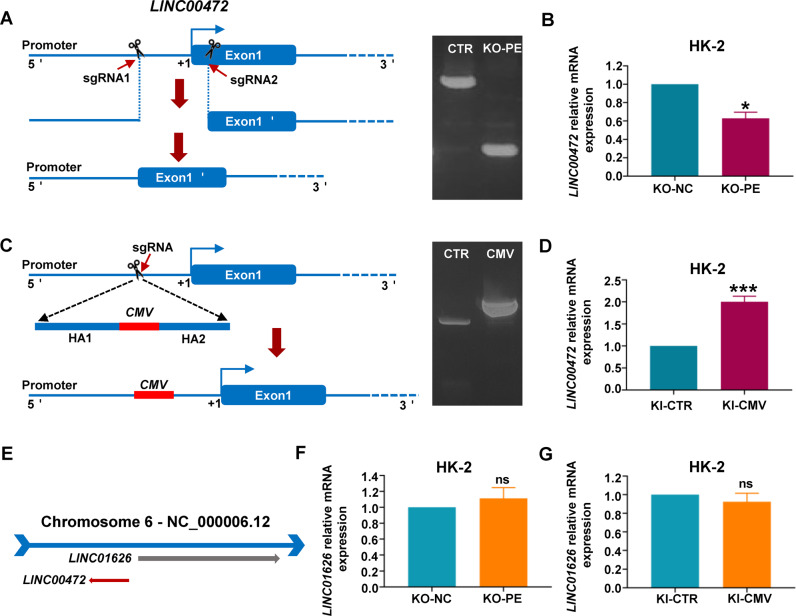
CRISPR-Cas9 was used to construct LINC00472 stable knockdown and overexpression cell lines. **A** Display diagram of knockdown shear site design. A pair of sgRNAs were designed upstream and downstream of the transcription starting site of LINC00472 gene (Left). DNA agarose electrophoresis showed successful cleavage (Right). **B** RT-qPCR was used to detect the inhibition efficiency of LINC00472 knockdown cell lines. **C** Display diagram of CMV knock-in design. sgRNA was designed in the promoter region of LINC00472, 1 kb homologous arm sequences upstream and downstream of the shear site were added on two sides of the CMV sequence (Left). DNA agarose electrophoresis showed the CMV sequence was successful inserted (Right). **D** The expression level of LINC00472 was detected by RT-qPCR after inserting CMV sequence. **E** Location diagram of LINC00472 and its adjacent gene LINC01626. RT-qPCR was used to detect the expression of adjacent gene LINC01626 after knockdown (**F**) or overexpression (**G**) of LINC00472 by CRISPR-Cas9. In **B**, **D**, **F**, and **G**, data were shown as mean ± SD of three independent experiments (*P < 0.05, ***P < 0.001, ns non-significant).

Next, we constructed cell lines with stable knockdown and overexpression of LINC00472 by using CRISPR-Cas9. Different from the knockout of common coding protein genes, the frameshift mutation caused by unit point shearing has low effective for lncRNA knockdown [22]. And not only that, due to the long length of LINC00472, we could not completely remove it. Therefore, we decided to reduce LINC00472 expression level by removing the sequence of its transcription starting site (TSS) to affect its transcription. We designed two shear sites that one is in the promoter region before the TSS and the other is in the first exon after the TSS (Fig. 4A). After monoclonal screening and sequencing, we obtained the successfully sheared cell line HK-2 KO-PE and the control cell line HK-2 KO-NC. RT-qPCR results showed the expression of LINC00472 in HK-2 KO-PE cells was indeed downregulated (Fig. 4B). To obtain the cell line with stable overexpression of LINC00472, we inserted a CMV enhancer sequence into the promoter region (Fig. 4C). Similarly, after monoclonal screening and sequencing, we obtained the cell line with successful sequence insertion cell line HK-2 KI-CMV and control cell line HK-2 KI-CTR, and the RT-qPCR results indicated that LINC00472 was successfully overexpressed in HK-2 KI-CMV cells (Fig. 4D). Inserting or deleting a certain sequence of a gene usually has a great risk of changing the expression of adjacent genes (Fig. 4E). In order to determine whether our scheme will affect the expression of genes near LINC00472, we checked the mRNA level of adjacent gene LINC01626 by using RT-qPCR, and the results showed that the mRNA level of LINC01626 did not change in both HK-2 KO-PE cell line and HK-2 KI-CMV cell line comparing with the control cell line (Fig. 4F, G).

### Overexpression of LINC00472 inhibited cell proliferation and enhanced intercellular connectivity

**Fig. 5:**
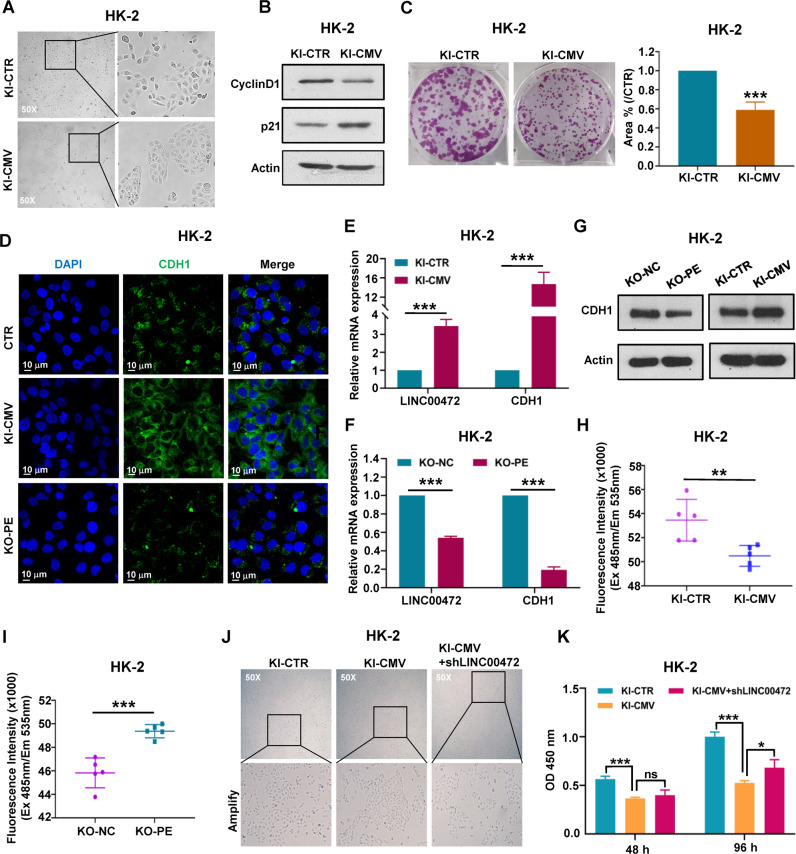
Overexpression of LINC00472 inhibited cell proliferation and enhanced intercellular connectivity. **A** The cell growth morphology of *LINC00472* overexpression cell line (HK-2 KI-CMV) and control cell line (HK-2 KI-CTR) were observed by fluorescence microscope. **B** Western blot was used to detect the levels of Cyclin D1 and p21 protein in HK-2 KI-CMV and HK-2 KI-CTR cell line. **C** Single cell clone formation assay of HK-2 KI-CMV and HK2 KI-CTR cell line (Left). Statistics of clone formation Area (Right). **D** The distribution of CDH1 were detected by immunofluorescence. The expression of CDH1 was detected by using RT-qPCR in HK-2 KI-CTR/HK-2 KI-CMV cell line (**E**) and HK-2 KO-NC/HK-2 KO-PE cell line (**F**). **G** Western blot was used to detect CDH1 protein level. **H**, **I** Monolayer cell permeability test is used to reflect the tightness of intercellular connections. The molecular permeability of LINC00472 high expression group was significantly lower than that of control group (**H**), while the molecular permeability of LINC00472 low expression group was evidently higher than control group (**I**). **J** HK-2 KI-CMV cell line was transfected with LINC00472 lentivirus interference vector (HK-2 KI-CMV/shLINC00472), and the cell growth morphology were observed by fluorescence microscope. **K** Cell Counting Kit-8 test was used to detect the proliferation of HK-2 KI-CTR, HK-2 KI-CMV, and HK-2 KI-CMV/shLINC00472 cell lines. In **C, E**, **F**, **H, I**, and **K**, data were shown as mean ± SD of three independent experiments (*P < 0.05, **P < 0.01, ***P < 0.001, ns non-significant).

By inserting a CMV sequence, we obtained a cell line with high expression of LINC00472, and then we found an obvious and interesting phenomenon that is the cell morphology of LINC00472 overexpressed cell line changed obviously compared with the control cell line, and the cells no longer grew in dispersion, but grew in clusters (Fig. 5A). In addition, the results of western blot detection of cell proliferation related proteins and single cell clone formation indicated that overexpression of LINC00472 significantly inhibited cell proliferation and reduced the ability of clone formation (Figs. 5B, C and S5A). Next, we detected the expression and distribution of CDH1 (also named E-cadherin), which could be used as a marker of intercellular adhesion. Immunofluorescence results indicated that the expression of CDH1 in HK-2 KI-CMV cells significantly increased and the distribution of CDH1 among cells was more aggregated comparing with the cells of control group; On the contrary, the expression of CDH1 decreased significantly in HK-2 KO-PE cells comparing with control group (Fig. 5D). Moreover, we also detected the mRNA and protein level of CDH1 using RT-qPCR and western blot, and the results showed that the level of CDH1 in LINC00472 high expression cell line was significantly higher than that in the control group, while it was the opposite in LINC00472 low expression cell line (Fig. 5E–G). In addition, we used monolayer cell permeability test to further reflect the degree of intercellular connectivity. The detection of FITC-Dextran fluorescence intensity showed that the cell permeability of LINC00472 high expression group was significantly lower than that of the control group, while the cell permeability of LINC00472 low expression group was distinctly higher than control group (Fig. 5H, I). The above experimental demonstrated that overexpression of LINC00472 could inhibit cell proliferation and significantly increase the intercellular connection.

Next, we transfected LINC00472 lentivirus interference vector in HK-2 stably overexpressing cell line HK-2 KI-CMV. Then we observed the growth state of the cells and detected the cell proliferation ability. The imaging results of cell growth morphology showed that when LINC00472 was inhibited, the degree of cell aggregation of HK-2 KI-CMV decreased obviously, which further indicated that LINC00472 affected the intercellular connection (Fig. 5J). In addition, CCK-8 results also showed that the proliferation ability of HK-2 KI-CMV cells was significantly reduced compared with the control cell line HK-2 KI-CTR, while after inhibiting the expression of LINC00472 in HK-2 KI-CMV cells, the cell proliferation ability was partially restored (Fig. 5K).

### LINC00472 is highly correlated with extracellular matrix and cell metastasis-related pathways

**Fig. 6:**
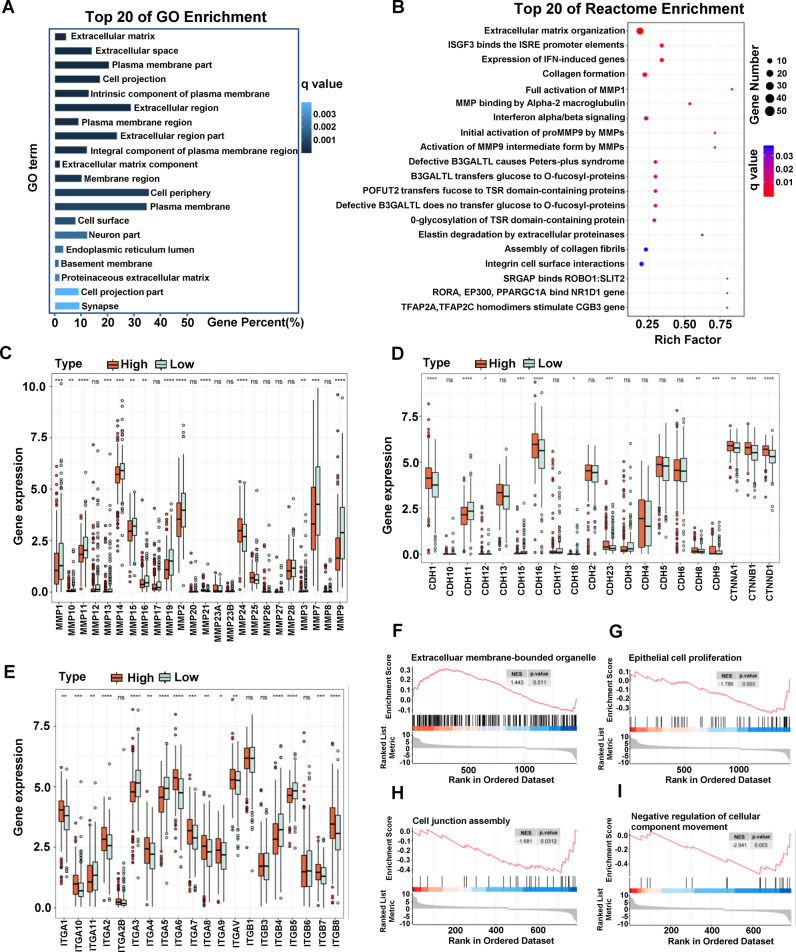
*LINC00472* is associated with extracellular matrix organization. **A** Top 20 of GO Enrichment of differentially expressed genes identified by RNA-Seq. **B** Top 20 of Reactome Enrichment of differentially expressed genes identified by RNA-Seq. **C** Relationship between the expression of LINC00472 and the level of MMPs family members in TCGA RCCC samples. **D** Relationship between LINC00472 and cadherin family members. **E** Relationship between LINC00472 and integrin subunit family members. GSEA showed the differences of extracellular membrane-bounded organelle (**F**), epithelial cell proliferation (**G**), cell junction (**H**), and negative regulation of cellular component movement (**I**) between high and low expression LINC00472 groups in TCGA RCCC samples.

To further analyze the function of LINC00472 in cells, we performed RNA sequencing (RNA-seq) on two pairs of cell lines (HK-2 KI-CTR and KI-CMV, HK2 KO-NC and KO-PE) constructed by CRISPR-Cas9. RNA-seq results indicated that the main item obtained from GO enrichment analysis is extracellular matrix (Fig. 6A). Reactome enrichment analysis also showed that the changed pathways mainly focus on the extracellular matrix organization and cell membrane, involving matrix metalloproteinases (MMPs) family activation, integrin cell surface interactions (Fig. 6B). Remarkably, most of the functions and pathways obtained by enrichment analysis are related to cell metastasis.

Next, we divided TCGA RCCC samples into high expression and low expression groups according to the median expression level of LINC00472, and then analyzed the expression levels of MMPs family members, integrin subunit family members, and cadherin family members in the two groups of samples. As shown in Fig. 6C, the expression level of most members of MMPs family in LINC00472 low expression group was higher than that in LINC00472 high expression group. Proteins in MMPs family are involved in the breakdown of extracellular matrix in normal physiological processes, such as embryonic development, reproduction, and tissue remodeling, as well as in cancer processes, especially cell metastasis. According to the analysis results, we speculated that the low expression of LINC00472 enhances the decomposition of extracellular matrix by MMPs family, and then promotes cell metastasis. In addition, the analysis of cadherin family proteins and integrin subunit family proteins showed that the expression level of most members of the two family in LINC00472 high expression group was higher than that in LINC00472 low expression group (Fig. 6D, E). These results further indicated that the high expression of LINC00472 may enhance the intercellular connection.

Besides, we calculated the differential genes of high and low expression groups and analyzed their functional enrichment. GO and KEGG analysis showed that the differentially expressed genes were still mainly enriched in the regulation of extracellular matrix and cell adhesion (Fig. S6A–C). These results are highly consistent with the analysis results of RNA-Seq. In addition, GSEA enrichment analysis showed that high expression of LINC00472 promoted the extracellular membrane-bounded organelle entries and inhibited the proliferation of epithelial cells, while low expression of LINC00472 reduced cell junction assembly and increased the regulation of cellular component movement (Fig. 6F–I).

### ITGB8 is a potential target gene regulated by LINC00472

**Fig. 7:**
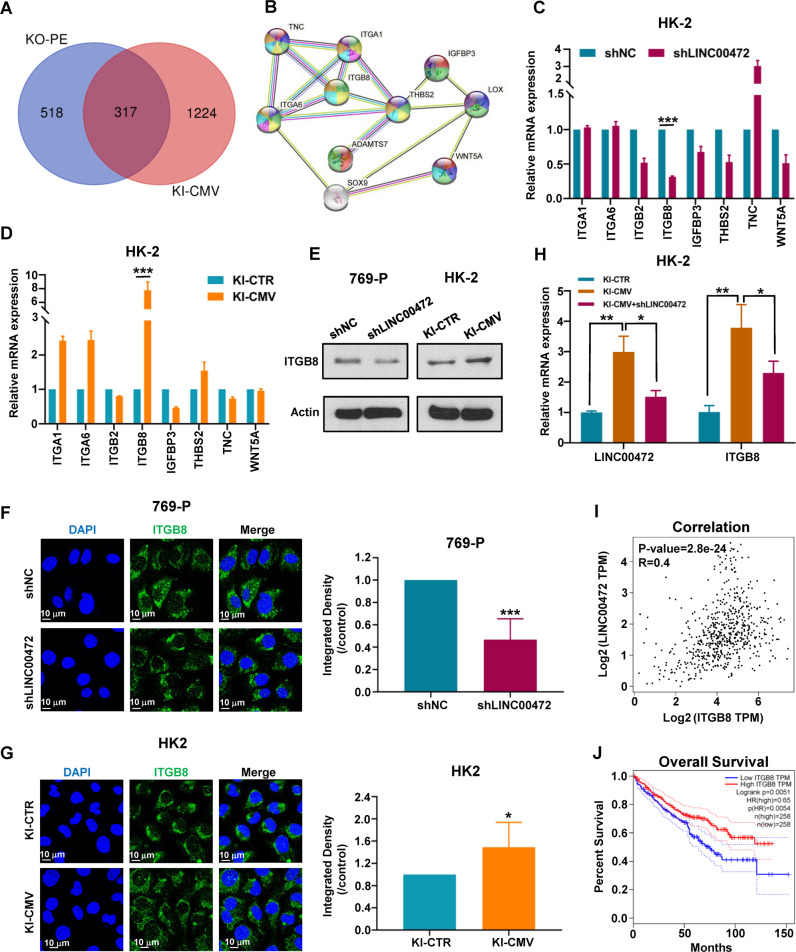
ITGB8 is a potential target gene regulated by LINC00472. **A** Analysis of RNA-seq overlap difference genes of HK-2 KO-NC/KO-PE and HK-2 KI-NC/KI-CMV. **B** The central sub network of the network which got by using PPI interaction analysis of differential genes produced by RNA-Seq in STRING database. RT-qPCR was used to detect the mRNA level of these network node molecules in HK-2 shNC and shLINC00472 cell line (**C**), and HK-2 KI-CTR and HK-2 KI-CMV cell line (**D**). **E** Western blot was used to detect the ITGB8 protein level in HK-2 and 769-P cells. The expression and distribution of ITGB8 in 769-P cell (**F**) and HK-2 cell (**G**) were detected by immunofluorescence (Left). Statistics of integrated density (Right). **H** RT-qPCR was used to detect the mRNA level of LINC00472 and ITGB8. **I** The correlation between LINC00472 and ITGB8. **J** Relationship between the expression of ITGB8 and the prognosis of RCCC patients. In **C**, **D**, **F**–**H**, data were shown as mean ± SD of three independent experiments (*P < 0.05, **P < 0.01, ***P < 0.001).

We analyzed the RNA sequencing data of HK-2 KO-NC/KO-PE and HK-2 KI-NC/KI-CMV, and found 317 genes with the same expression level trend (Fig. 7A). Through PPI interaction analysis of the 317 genes in STRING database, we found a sub network located at the hub of the network, suggesting that these network node molecules may play an important role in the function of LINC00472 (Fig. 7B). Next, we detected the expression level of these network node molecules by using RT-qPCR, and the results showed that the expression of integrin family protein ITGB8 significantly decreased after LINC00472 was inhibited (Figs. 7C and S7A), and distinctly increased when LINC00472 was overexpressed (Fig. 7D). Western blot and immunofluorescence results were consistent with the results of RT-qPCR (Fig. 7E–G). In addition, we suppressed LINC00472 expression in LINC00472 overexpressed cell line, and then examined the expression level of ITGB8. As shown in Fig. 7H, the mRNA level of ITGB8 decreased with the inhibition of LINC00472 in HK-2 KI-CMV cell line. Based on these results, we speculated that ITGB8 may be a potential target gene regulated by LINC00472. We analyzed the correlation between LINC00472 and ITGB8 in RCCC and normal tissues on GEPIA website. The results showed that the Spearman correlation coefficient between LINC00472 and ITGB8 was 0.4, indicating that there was a correlation between them (Fig. 7I). Furthermore, we also analyzed the relationship between the expression of ITGB8 and the prognosis of RCCC patients. As shown in Fig. 7J, the prognosis of patients with low expression of ITGB8 was significantly worse than that of patients with high expression of ITGB8, showing the same trend as LINC00472.

### LINC00472 may interact with P300 to regulate histone modification

**Fig. 8:**
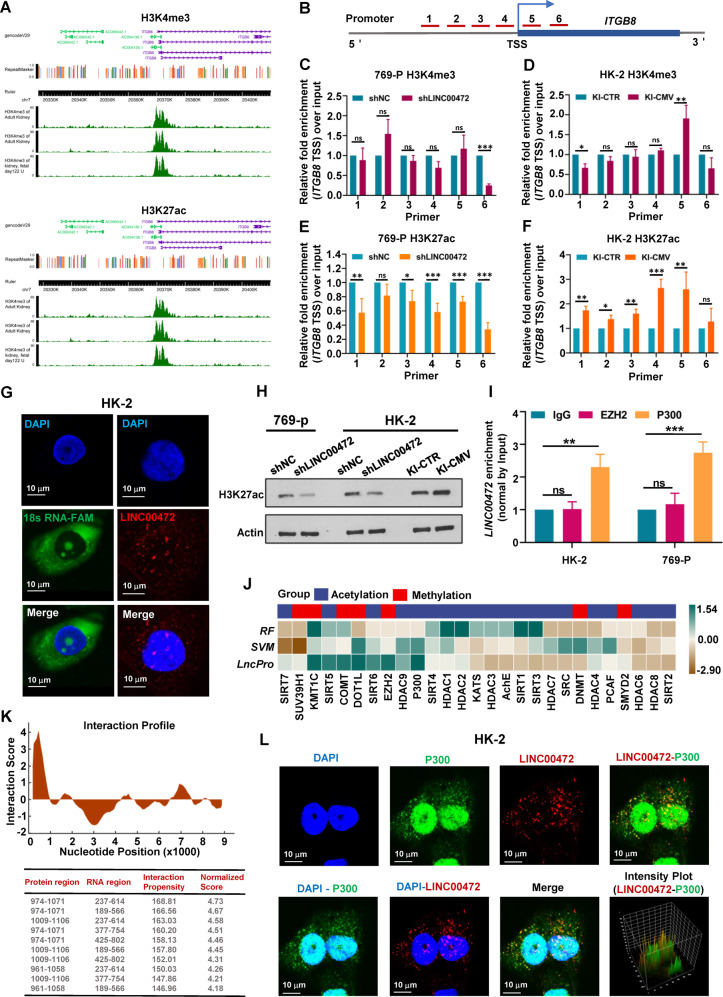
LINC00472 regulates H3K27 acetylation at the ITGB8 transcription start site. **A** Two significantly enriched histone modification peaks at the ITGB8 transcription start site in the ChIP-Seq database that is H3K4me3 (Top) and H3K27ac (Bottom). **B** Six pairs of primers were designed near the TSS site of ITGB8. **C**–**F** Chromatin immunocoprecipitation was used to detect the level of histone modification. Inhibition of LINC00472 expression significantly decreased the level of H3K27ac near ITGB8 TSS site (**E**), while the level of H3K4me3 not (**C**). Overexpression of LINC00472 distinctly increased the level of H3K27ac near ITGB8 TSS site (**F**), but the level of H3K4me3 not (**D**). **G** RNA FISH results showed that LINC00472 was distributed both in the nucleus and cytoplasm. **H** The total H3K27ac levels in 769-P and HK-2 cells were detected by Western Blot. **I** RNA immunocoprecipitation was used to verify the interaction between LINC00472 and EZH2 or P300. **J** Using the algorithm support provided by RPISeq and lncPro database to analyze the possibility of interaction between histone modification participants and LINC00472. **K** Prediction of interaction sites between LINC00472 and P300. **L** RNA FISH and immunofluorescence showed that LINC00472 and P300 had multiple overlaps in cells. In **C**–**F** and **I**, data were shown as mean ± SD of three independent experiments (*P < 0.05, **P < 0.01, ***P < 0.001, ns non-significant).

Next, we want to explore how LINC00472 regulates the expression of ITGB8. It is noteworthy that there are two obviously enriched histone modification peaks at the ITGB8 transcription start site (TSS) in the ChIP-Seq database that is H3K27ac and H3K4me3 (Figs. 8A, S8A, B). Then, we designed six pairs of primers on upstream and downstream of ITGB8 TSS, and detected the levels of H3K27ac and H3K4me3 by using Chromatin Immunoprecipitation. The results showed that inhibition of LINC00472 significantly reduced the level of H3K27ac near the ITGB8 TSS (Figs. 8E and S8D), while the level of H3K4me3 did not change (Figs. 8C and S8C). Conversely, overexpression of LINC00472 obviously increased the level of H3K27ac near the ITGB8 TSS, while H3K4me3 not (Fig. 8D, F). According to the results of chromatin immunoprecipitation, we speculate that LINC00472 is involved in the regulation of histone acetylation modification.

Subsequently, we analyzed the distribution of LINC00472 by RNA fluorescence in situ hybridization (FISH) and found that it was distributed both in the nucleus and cytoplasm, which providing a spatial possibility to participate in the regulation of histone modification (Fig. 8G). We checked the total level of H3K27ac in HK-2 and 769-P cells using western blot and the results showed that inhibition of LINC00472 significantly reduced the level of H3K27 acetylation; On the contrary, overexpression of LINC00472 obviously increased the level of H3K27 acetylation (Fig. 8H). RPISeq provides two different methods (random forest, RF; support vector machine, SVM) to evaluate the possibility of protein binding to RNA. Using the algorithm support provided by RPISeq (http://pridb.gdcb.iastate.edu/RPISeq/) and lncPro (http://bioinfo.bjmu.edu.cn/lncpro/) database, we analyzed the possibility of interaction between histone modification participants and LINC00472. The results showed that LINC00472 had the highest correlation with P300 (Fig. 8J), a protein functions as histone acetyltransferase that regulates transcription via chromatin remodeling and is important in the processes of cell proliferation and differentiation [23]. Notably, RNA immunoprecipitation results showed that P300 antibody enriched more LINC00472 RNA comparing with negative control IgG and EZH2 antibody, which indicated that P300 and LINC00472 may be combined with each other (Fig. 8I). Moreover, by analyzing the sequence and structure of LINC00472 and P300, we obtained some potential interaction sites between them (Fig. 8K). In addition, we detected the intracellular localization of LINC00472 and P300 by RNA FISH and immunofluorescence, and the results displayed that LINC00472 and P300 had multiple overlaps in the cell (Fig. 8L). According to the results of RNA immunoprecipitation and immunofluorescence, we speculated that LINC00472 may interact with P300, and then participate in the regulation of histone acetylation in cells.

## Discussion

RCCC is difficult to diagnose in the early stage and easy to metastasize [24]. Finding effective clinical diagnostic markers and molecular therapeutic targets is crucial to improve the therapeutic effect of RCCC. Noncoding RNA has been a hot research field in recent years, especially its functions related to cancer, neurodegenerative diseases and immune related diseases [25–27]. Benefit by the non-conservation of noncoding RNA, it is easier for researchers to find specific molecular targets corresponding to a certain disease [28]. Here, we found a lncRNA LINC00472, which decreased significantly in RCCC and showed a downward trend with the progress of cancer stage, also has a highly correlated with the prognosis of patients.

LINC00472 has been shown to promote apoptosis and inhibit cell proliferation by regulating miR-24-3p/DEDD in lung adenocarcinoma [29, 30]. Similarly, LINC00472 was found to promote cell apoptosis and suppresses cell proliferation through elevating PDCD4 expression by sponging miR-196a in colorectal cancer [31]. There were also studies have shown that ERα can increase the level of LINC00472 and then inhibit the phosphorylation of NF-κB in breast cancer [32, 33]. In our study, we found that inhibition of LINC00472 promoted cell proliferation and migration, which are consistent with previous studies. In addition, we found that overexpression of LINC00472 could significantly enhance intercellular adhesion. RNA-seq and TCGA enrichment analysis showed that LINC00472 was mainly involved in extracellular matrix pathway, MMPs family and integrin family protein regulation, which provides new evidence for LINC00472 to inhibit cell migration.

For the molecular mechanism, most early studies confirmed an important function of LINC00472, that is it can act as “sponge” to competitively bind miRNA to regulate the expression of target genes [13, 34, 35]. We speculate that this function is related to its own sequence structure, that is, longer sequences provide more potential binding sites for miRNA [36]. Here, we found a potential target gene of LINC00472 through transcriptome sequencing and STRING analysis, and we verified it through RT-qPCR and Western Blot. By studying the mechanism of how LINC00472 regulates target gene, we found that the change of LINC00472 affected the acetylation level of H3K27 site in cells, and we speculate that this effect is likely to be generated through the interaction with acetyltransferase P300. In general, we found that LINC00472 plays an important role in the proliferation and metastasis of renal clear cells, and we preliminarily verified that it may participate in the regulation of histone modification. Bioinformatics analysis also indicates that it may be a potential prognostic diagnostic marker.

There are still many sections for further improvement and proof in our research. For example, the efficiency of knockdown of LINC00472 using CRISPR-Cas9 method needs to be further improved. Mechanistically, more direct evidence proving the interaction between LINC00472 and P300 needs further exploration, and how to regulate the acetylation of H3K27 site also needs further experiments to verify.

## Materials and methods

### Weighted gene co-expression network analysis

R package “WGCNA” was used to perform weighted gene co-expression network analysis (WGCNA) using The Cancer Genome Atlas (TCGA) RCCC expression matrix (FPKM). To build a scale-free network and calculate network topology matrix, a gene expression matrix was weighted by a soft threshold. We used a dimension reduction algorithm to visualize the network module composed of co-expressed genes in RCCC samples after clustering with a dynamic cut tree algorithm and merging similar modules. The survival data of patients in TCGA are used for module correlation analysis, and the correlation between corresponding module genes and survival was visualized.

### Publicly available mRNA data and ChIP-Seq data

Our study incorporated data from two publicly available datasets. TCGA RNA-Seq data (FPKM value) of samples from patients with RCCC (Illumina HiSeq 2000) were acquired from Genomic Data Commons (GDC) (http://portal.gdc.cancer.gov. accessed on 23 April 2021). GSE40435 reported the whole genome expression data of tumor and adjacent non tumor renal tissues of 101 pairs of RCCC patients [37]. We obtained the data from GEO (https://www.ncbi.nlm.nih.gov/geo. accessed on 16 April 2021), and then further analyzed the microarray data according to the expression of LINC00472. The ChIP-seq data used in this project comes from the new WashU Epigenome Browser (http://epigenomegateway.wustl.edu/browser). We analyzed all renal tissue data in roadmap data from GEO and visualized it using this website.

### Biological Process and Pathway Enrichment Analysis

DEGs between high- and low-LINC00472 expression groups were identified by using R package “DESeq2” [38]. Different pathways and items were identified between the two expression groups using Gene Ontology (GO), the Kyoto Encyclopedia of Genes and Genomes (KEGG) and Reactome enrichment analysis. In addition, we used GSEA to calculate dynamical scores for different enrichment items of high- and low-expression groups [39].

### Cell culture and construction of stable cell lines

HK-2 cells and 769-P cells were purchased from National Collection of Authenticated Cell Cultures (Shanghai, China). HK-2 cells were grown in DMEM/F12 (1:1) Medium with 10% fetal bovine serum (Gibco, California, USA). 769-P cells were cultured in RPMI 1640 Medium with 10% fetal bovine serum. All medium were supplemented with 10 U/ml of penicillin-streptomycin and all cells were cultured in a 5% CO2 humidified incubator at 37 °C. The control shRNA and Lentivirus-based LINC00472-targeting shRNA vectors were purchased from GENECHEM (Shanghai, China). The sequence of shLINC00472#1 is “GCAACAGAAGTATGTGCAA”, shLINC00472#2 is “GCCAGTATATACTGAACAT”. HK-2 cells and 769-P cells were transiently transfected with these vectors and screened by puromycin at a concentration of 2 μg/ml to generate stable monoclonal cell lines.

### CCK-8 and cell counts

Cell counting kit-8 (TRANS, FC-101) was used to detect cell proliferation according to the manufacturer’s instructions. Cell numbers was calculated by using Cell counter Scepter sensors 60 mm (Millipore, PHCC60050).

### Real-time quantitative PCR analysis

Real-time quantitative PCR (RT-qPCR) analysis was performed as previously described [40]. Total RNA was isolated using AG RNAex Pro Reagent (AG21101) according to the manufacturer’s protocol. Real-time quantitative PCR was performed using the TransScript All-in-One First-Strand cDNA Synthesis SuperMix for qPCR (One-Step gDNA Removal) (TRANS, AT341-01) and the PerfectStart SYBR Green qPCR SuperMix (TRANS, AT601-01). All the level of mRNAs were measured and normalized to β-actin, and the primers used are listed in Supplemental Table S1.

### Construction and transfection of CRISPR-Cas9 plasmid

The sgRNAs were designed from E-CRISPR (http://www.e-crisp.org/E-CRISP/), and the CRISPR-Cas9 plasmids and control vectors were synthesized in GenePharma (Shanghai, China). Plasmid transfection was performed with Lipofectamine 3000 reagent (Invitrogen, USA) according to the manufacturer’s instructions. After 24 h of transfection, added 2 μg/ml puromycin and cultured for two days, then diluted the surviving cells and transferred to 96 well plate for single cell screening. PCR and sequencing were used to identify whether the cells were successfully constructed.

### RNA sequencing

Total RNA was isolated using AG RNAex Pro Reagent (AG21101) according to the manufacturer’s protocol. The purification, quality inspection and PCR library construction of samples were completed by GENE DENOVO (Guangzhou, China). Differentially expressed genes were identified using the “DESeq2” package with standard settings. Genes with a *p* adjust <0.05 were considered differentially expressed, which were used for GO and KEGG enrichment analysis.

### Western Blotting

Western blotting was performed as previously described [41]. The antibodies used for western blotting include P27 (CST, 3686T), CDK4 (CST, 12790S), CyclinB1 (CST, 12231S), P21 (CST, 2947T), CyclinD1 (CST, 2978S), E-cadherin (Proteintech, 60335-01), ITGB8 (Abcam, ab243023), H3K27ac (Abcam, ab4729) and β-actin (CST, 8480S).

### Cell permeability assay

The cells were planted in the transwell chamber placed in the well plate. After the cells were full, removed culture medium, washed with PBS, then added new culture medium containing 5 μg/ml FITC-Dextran (10 KD), incubated in dark for 30 min, then collected the lower layer solution. The fluorescence intensity was detected by using multifunctional microplate reader (Beckman) at the wavelength of Ex 485 nm/Em 535 nm.

### Immunofluorescence microscopy and FISH

Immunofluorescence was performed as previously described [42]. The cells were seeded into a well plate covered with glass slides. At the treated time, the cells were fixed in 4% formaldehyde for 10 min at room temperature, washed two times in PBS, then permeabilizated with 0.5% Triton-X 100 for 10 min at 4 °C, and blocked with 3% BSA for 45 min at room temperature, followed by incubation for 1 h at room temperature with indicated antibodies. Washed 5 min each time for 3 times, and then incubated for 45 min with Alexa Flour secondary antibody (diluted 1:250 in blocking buffer). For RNA FISH, cells were fixed in 4% formaldehyde for 10 min at room temperature, washed two times in PBS, then permeabilizated with 0.5% Triton-X 100 for 10 min at 4 °C, followed by incubation overnight at 37 °C with LINC00472 specific probe (GenePherma, China). Cell images were obtained with confocal microscope (Olympus FV1000).

### RNA immunocoprecipitation

RNA immunocoprecipitation was performed as previously described [43, 44]. Briefly, cells were harvested and lysed with polysome lysis buffer (5 mM MgCl2, 10 mM KCl, 10 mM HEPES pH 7.0, 1 mM DTT, 0.5% NP-40, 100 U/ml RNase inhibitor, 20 μl/ml protein inhibitor calculator). Then lysed cells were incubated with 5 μg EZH2 (Abcam, ab191250) or P300 (Abcam, ab14984) antibody overnight at 4 °C. Next, using polysome lysis buffer to wash the non-specifically binding antibodies and incubated with Dynabeads protein G (Invitrogen, USA) for 4–6 h at 4 °C. Then washed and precipitated the RNA with ethanol and sodium acetate. The RNA fragments were reverse transcribed and analyzed by RT-qPCR.

### Chromatin immunoprecipitation (ChIP)

ChIP assay was detected by using SimpleChIP® Enzymatic Chromatin IP Kit (Magnetic Beads) according to the manufacturer’s instructions (CST, 9003S). The ChIP grade antibodies include H3K4me3 (Abcam, ab8580), H3K27ac (Abcam, ab4729) and the IgG negative control of SimpleChIP® Enzymatic Chromatin IP Kit. The primers used for RT-qPCR analysis are listed in Supplemental Table S2.

### Statistical analysis

Statistical analysis was performed using GraphPad Prism 8.0 version. Student’s t-test was used to make a statistical comparison between groups. *P* < 0.05 was considered statistically significant. Results were representative of at least three experiments under identical conditions.

## Supplementary information

Supplementary Figure S1

Supplementary Figure S2

Supplementary Figure S3

Supplementary Figure S4

Supplementary Figure S5

Supplementary Figure S6

Supplementary Figure S7

Supplementary Figure S8

Supplementary Figure Legends

Supplementary Tables

Original western blots

## Data Availability

The data sets generated and collected during this study will be available from the corresponding author upon reasonable request.
