# Supplementary figures and images for: LINC00472 inhibits cell migration by enhancing intercellular adhesion and regulates H3K27ac level via interacting with P300 in renal clear cell carcinoma
Source: Cell Death Discov. 2022 Nov 12;8:454. doi: 10.1038/s41420-022-01243-7 (PMC9653443; doi:10.1038/s41420-022-01243-7)

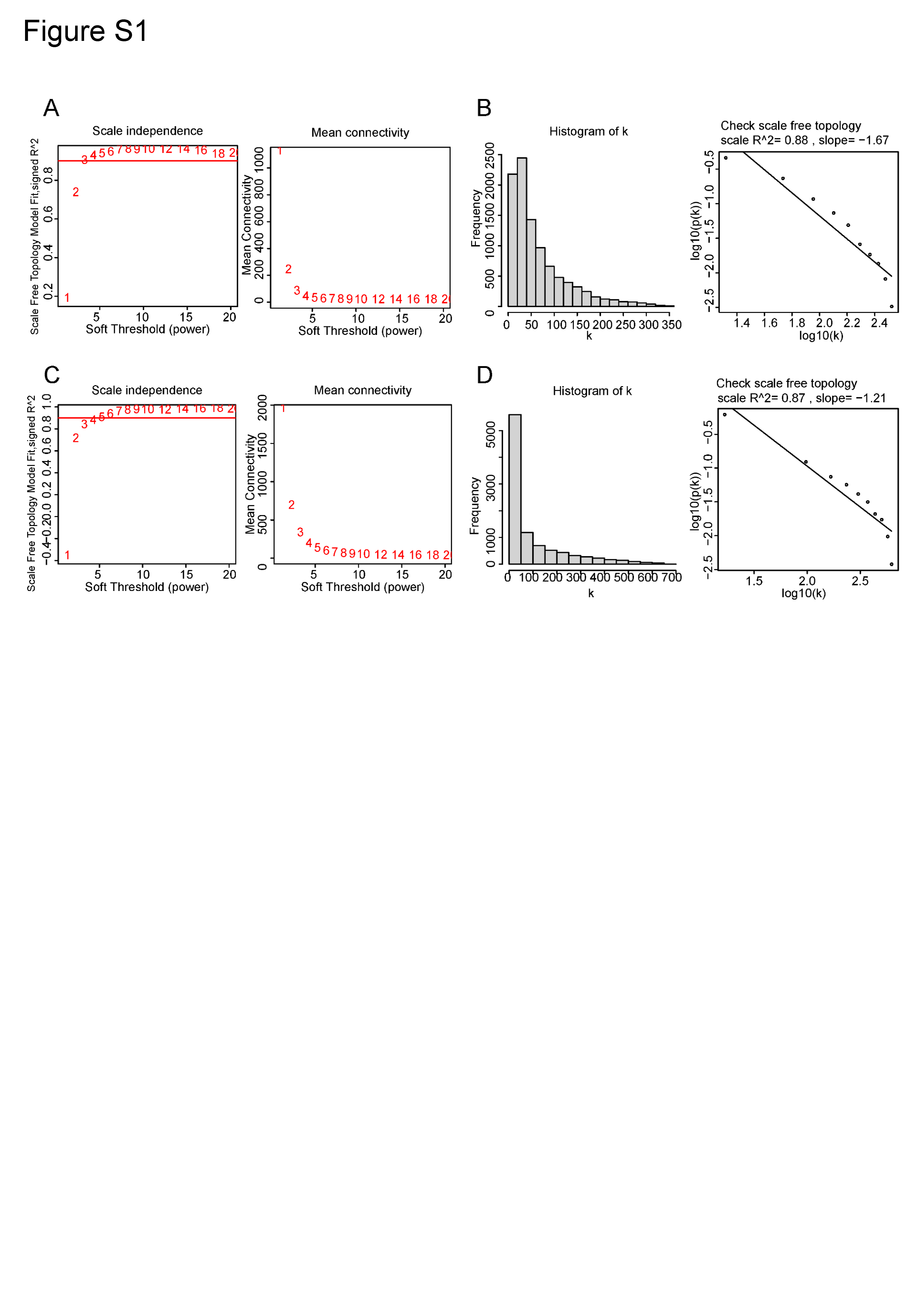

Supplement: Supplementary file 1 — Supplementary Figure S1 [file 41420_2022_1243_MOESM1_ESM.tif]

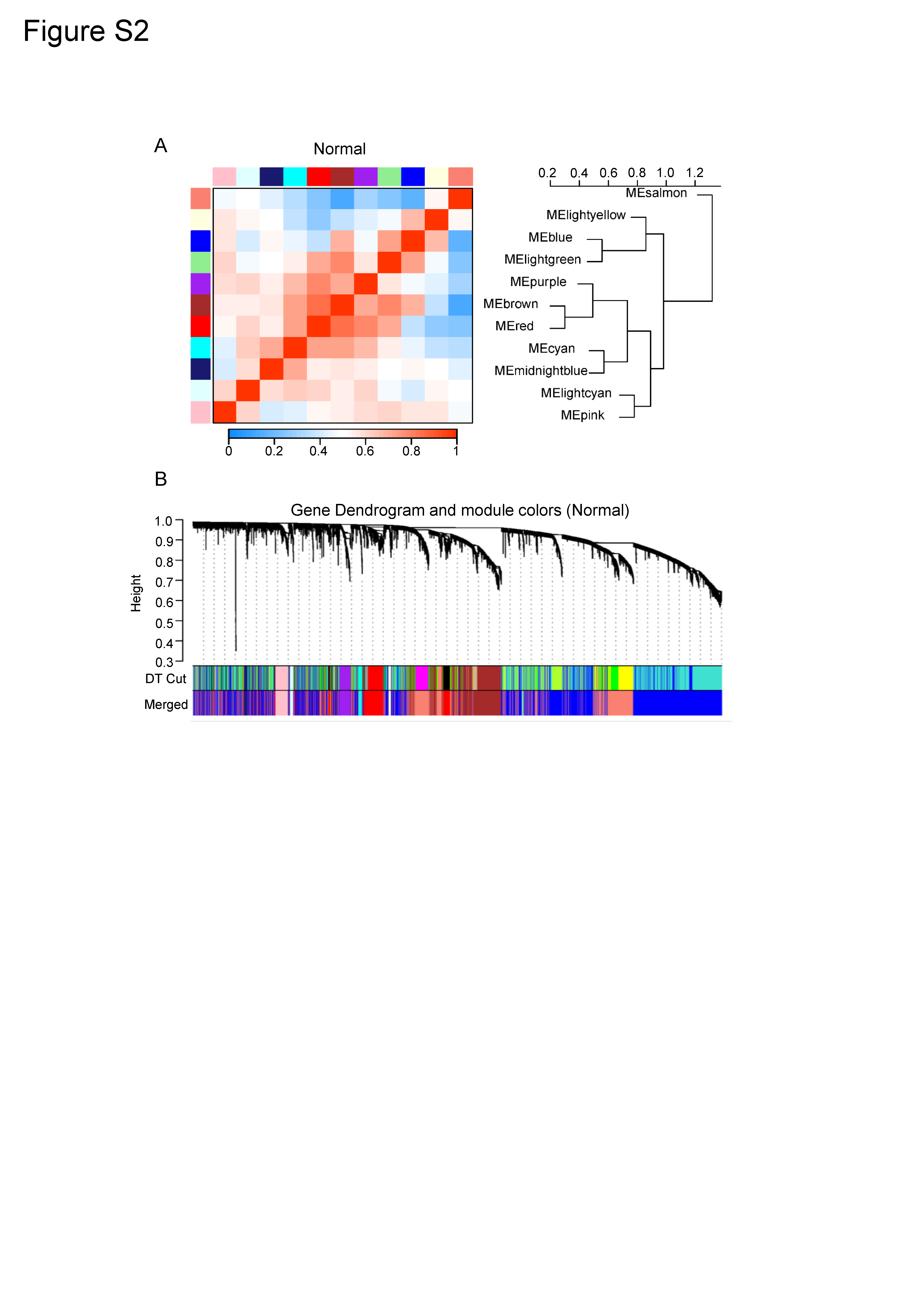

Supplement: Supplementary file 2 — Supplementary Figure S2 [file 41420_2022_1243_MOESM2_ESM.tif]

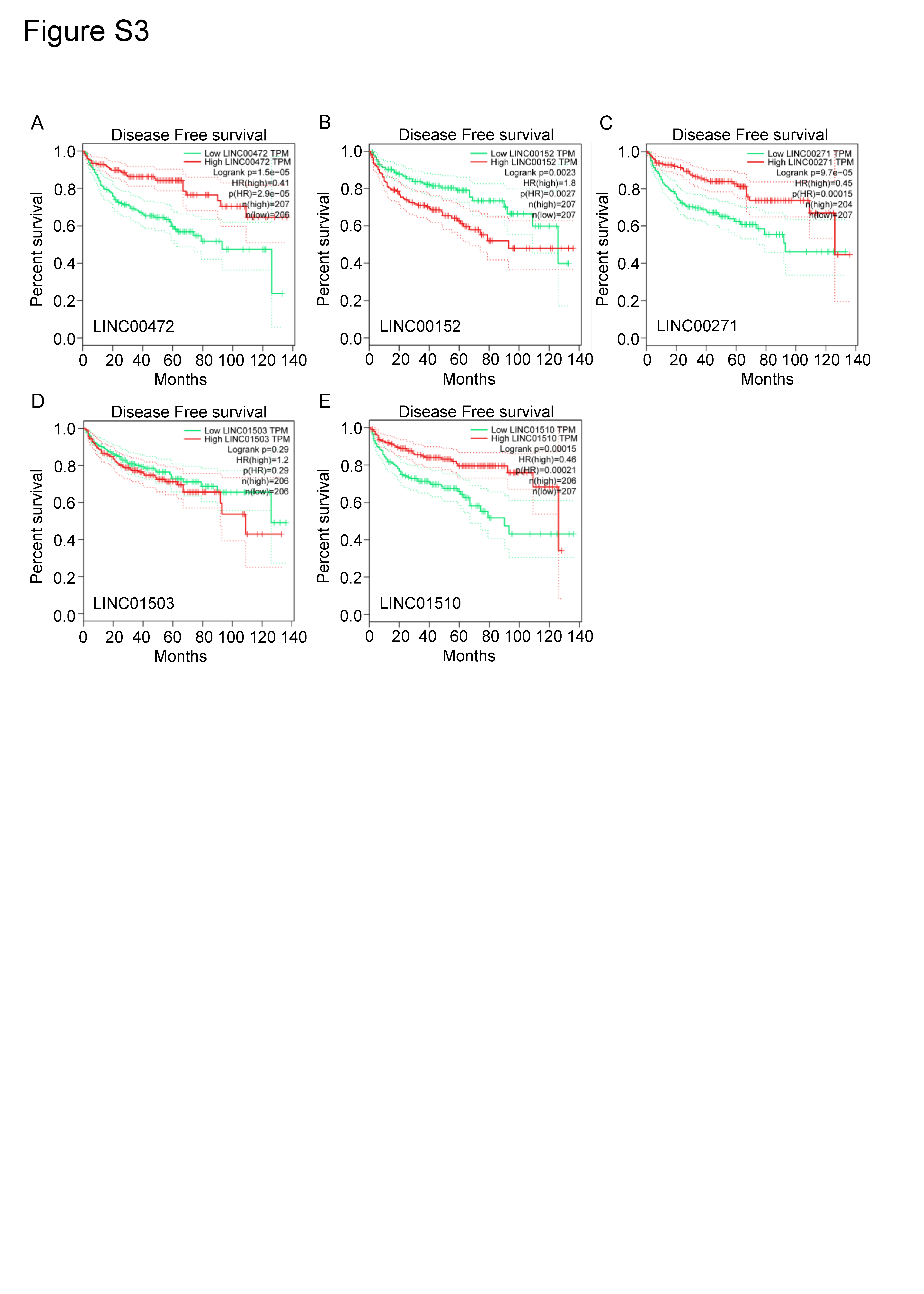

Supplement: Supplementary file 3 — Supplementary Figure S3 [file 41420_2022_1243_MOESM3_ESM.tif]

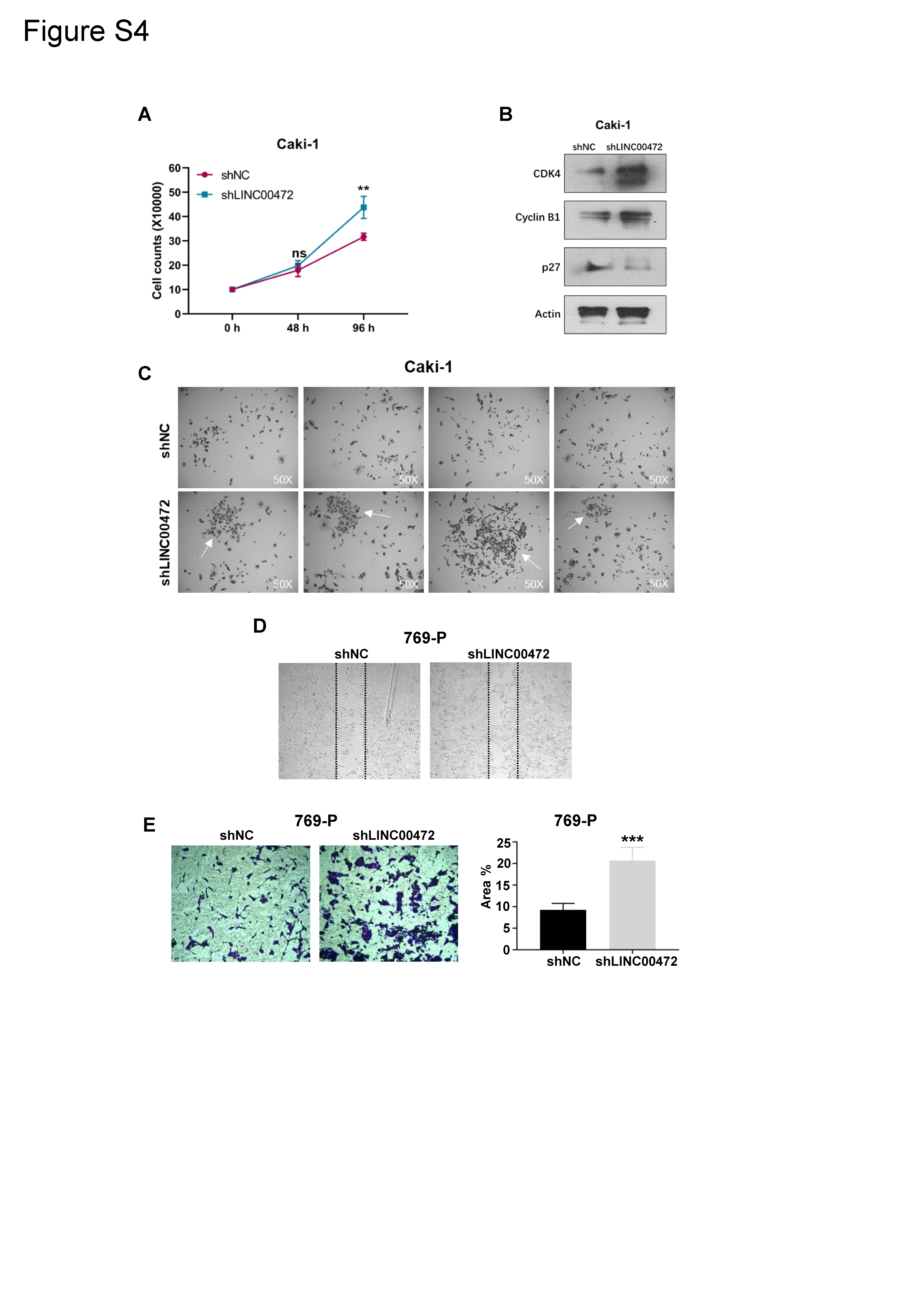

Supplement: Supplementary file 4 — Supplementary Figure S4 [file 41420_2022_1243_MOESM4_ESM.tif]

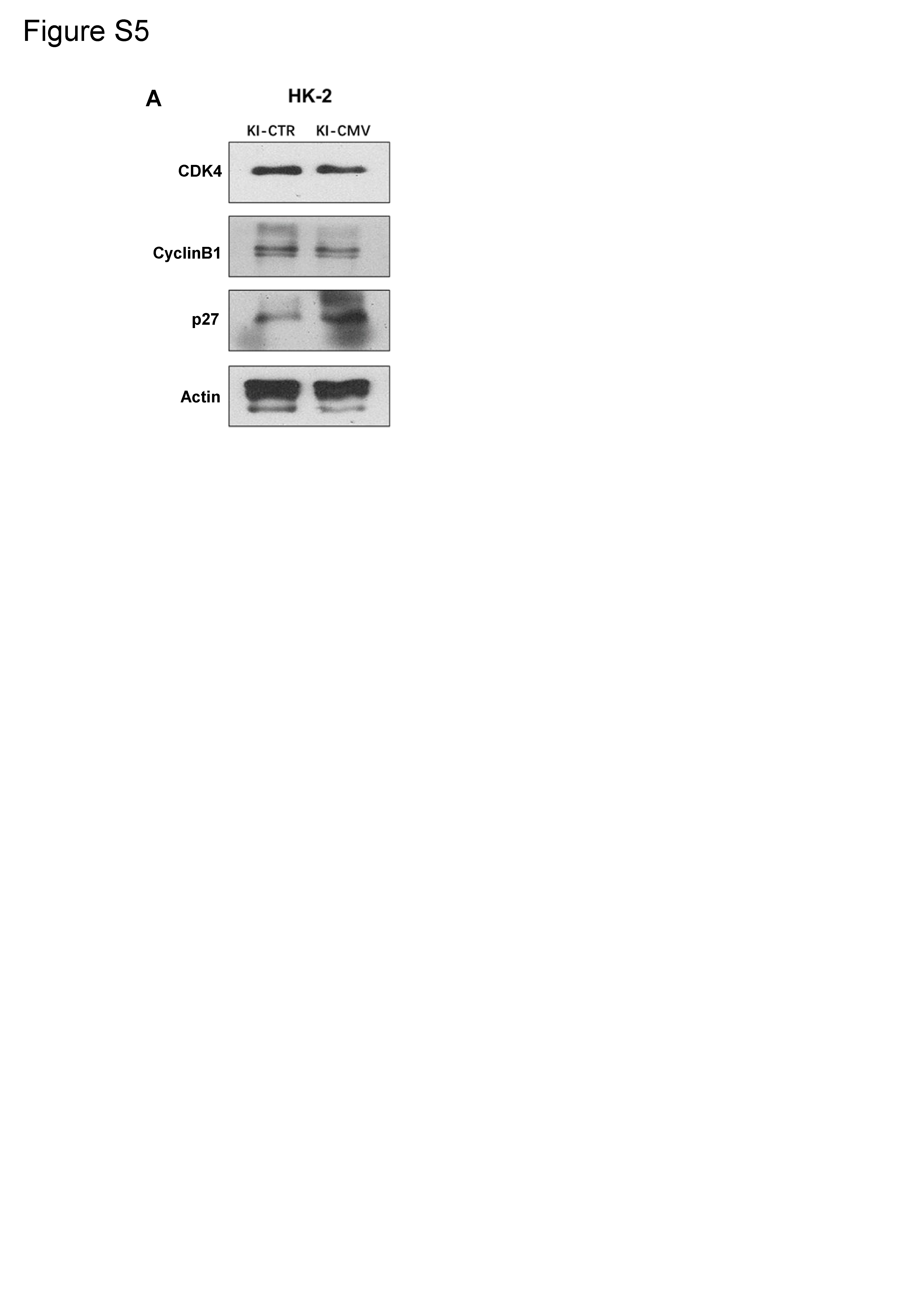

Supplement: Supplementary file 5 — Supplementary Figure S5 [file 41420_2022_1243_MOESM5_ESM.tif]

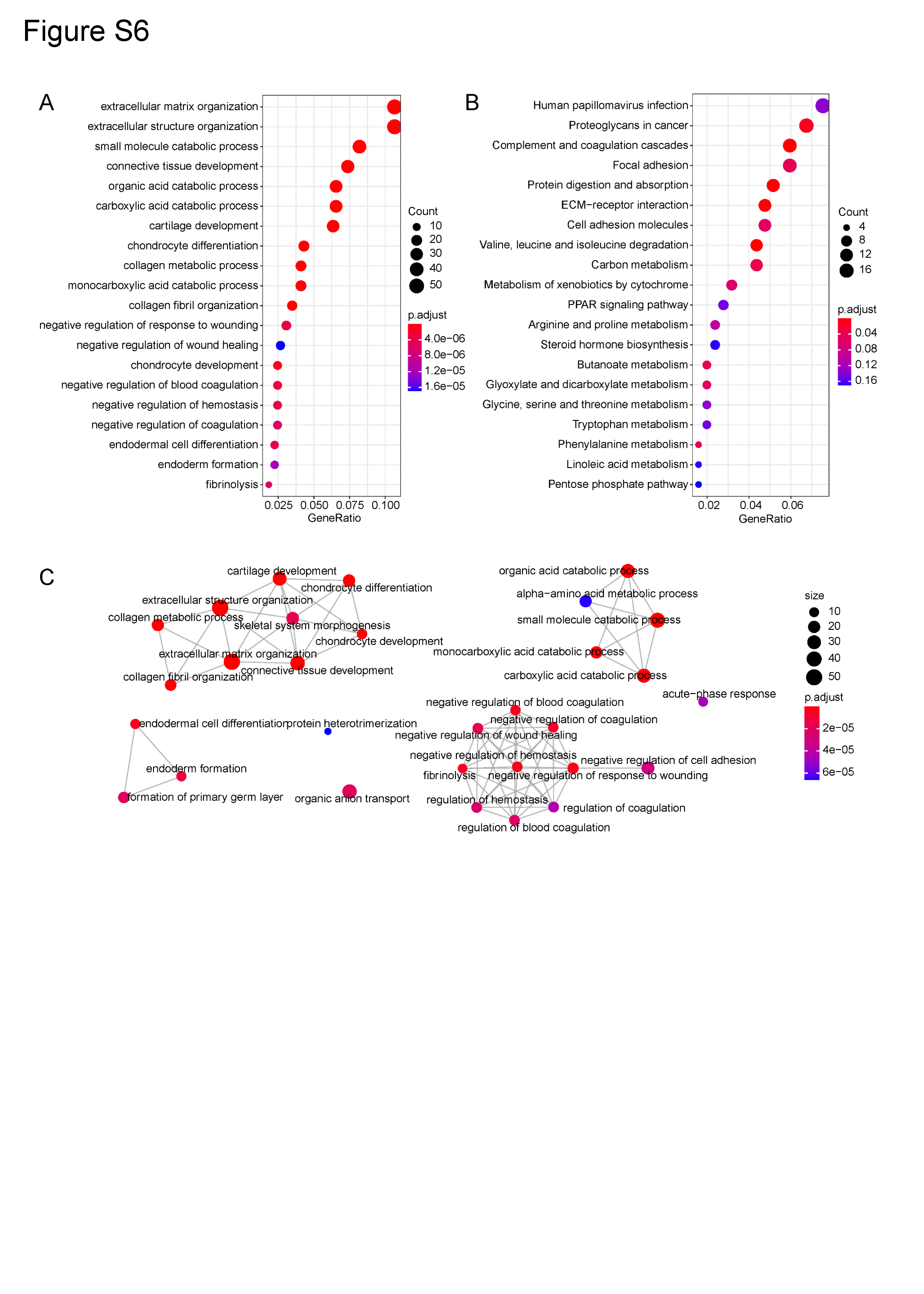

Supplement: Supplementary file 6 — Supplementary Figure S6 [file 41420_2022_1243_MOESM6_ESM.tif]

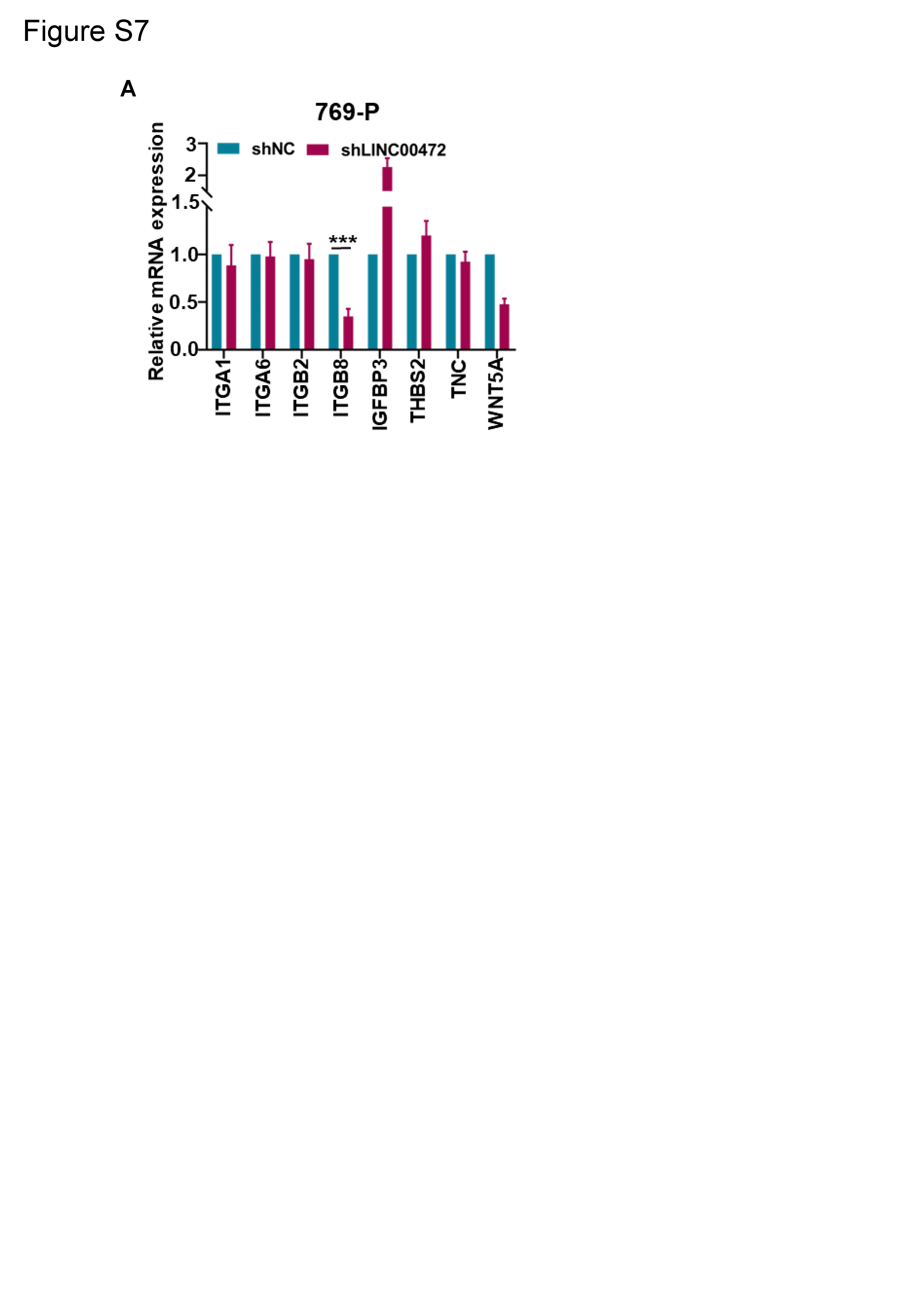

Supplement: Supplementary file 7 — Supplementary Figure S7 [file 41420_2022_1243_MOESM7_ESM.tif]

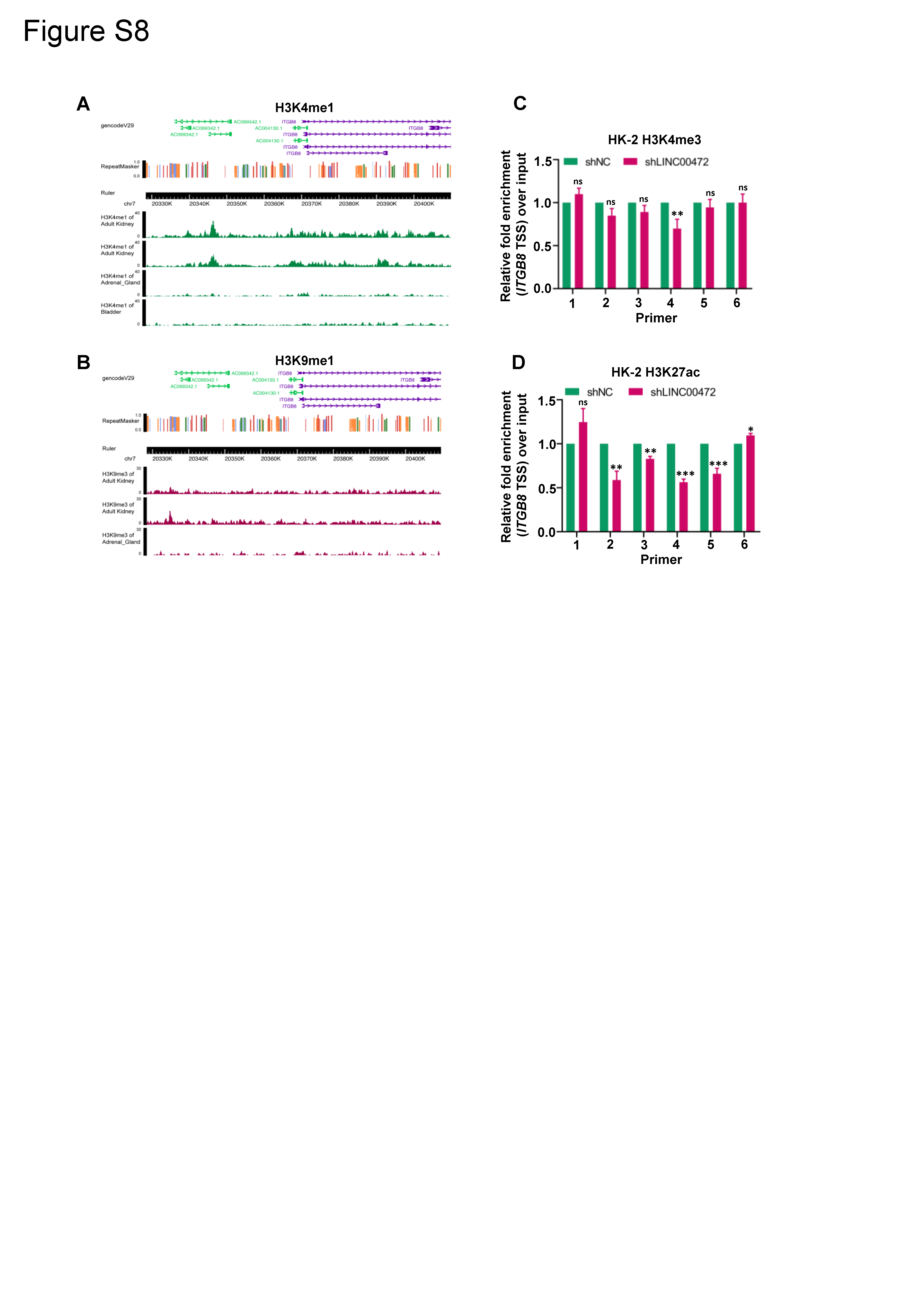

Supplement: Supplementary file 8 — Supplementary Figure S8 [file 41420_2022_1243_MOESM8_ESM.tif]

**Figure 3I**

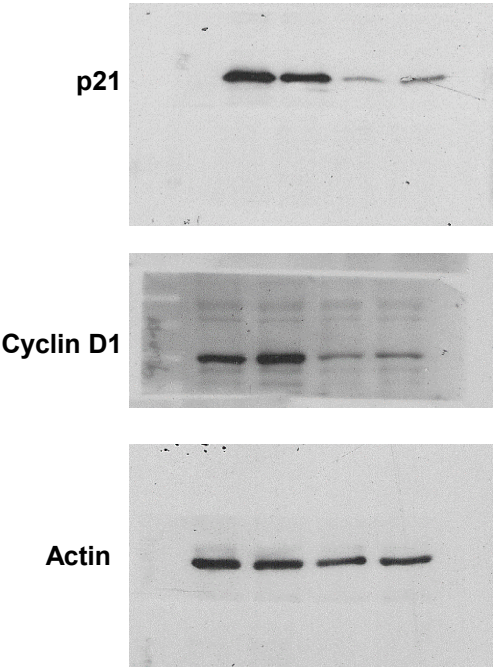

**Figure 5B**

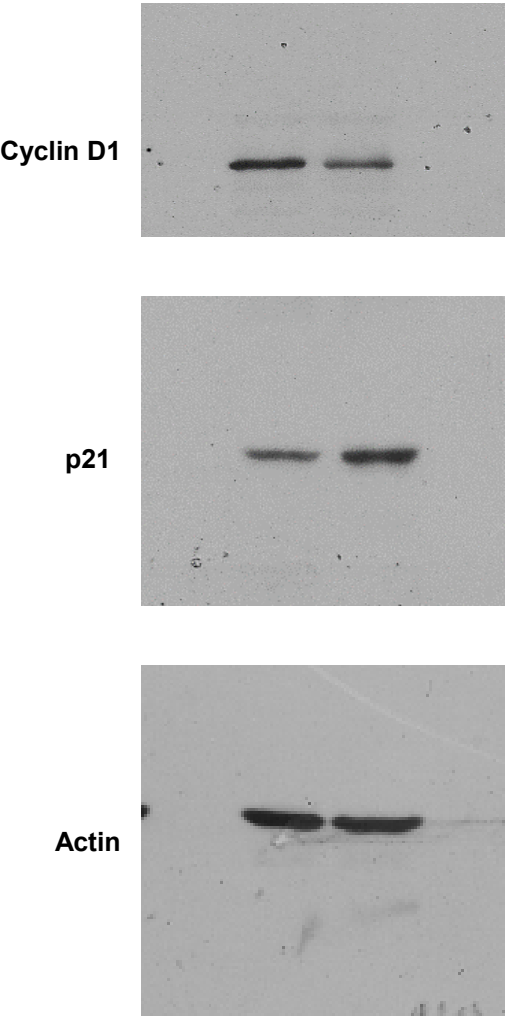

**Figure 5G**

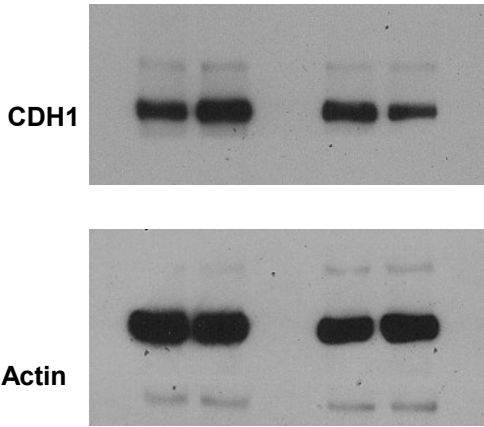

**Figure 7E**

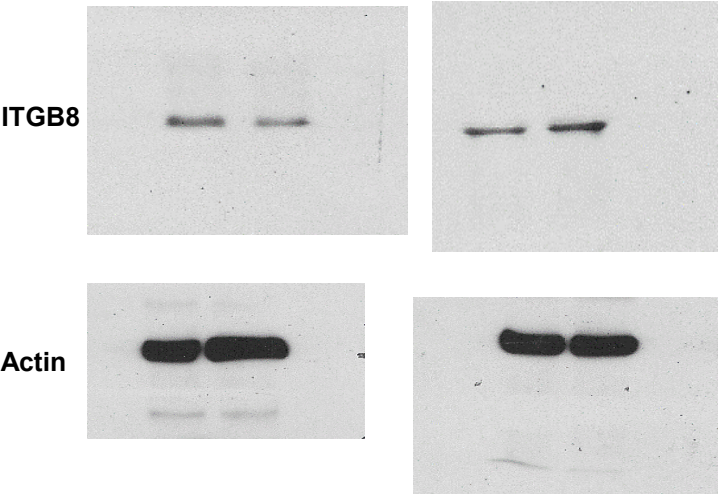

**Figure 8H**

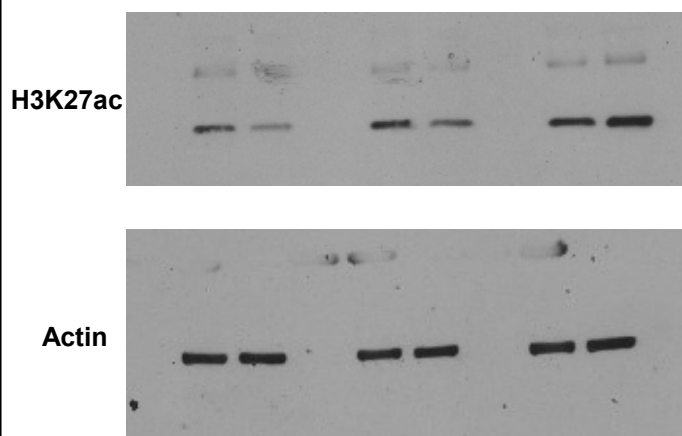

**Figure S4B**

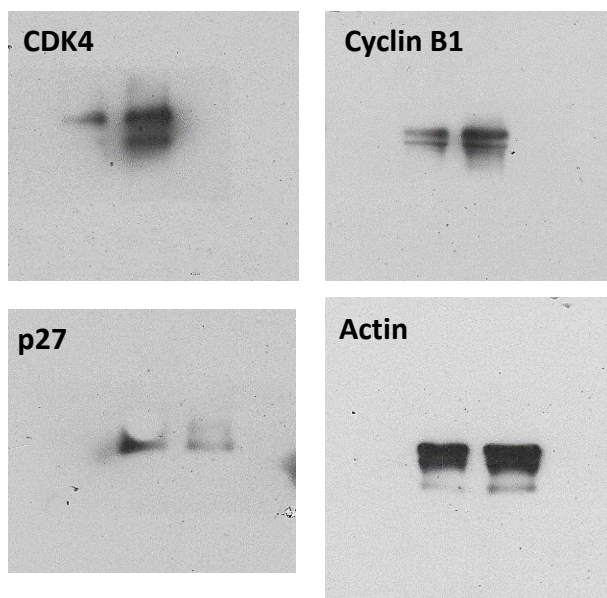

**Figure S5A**

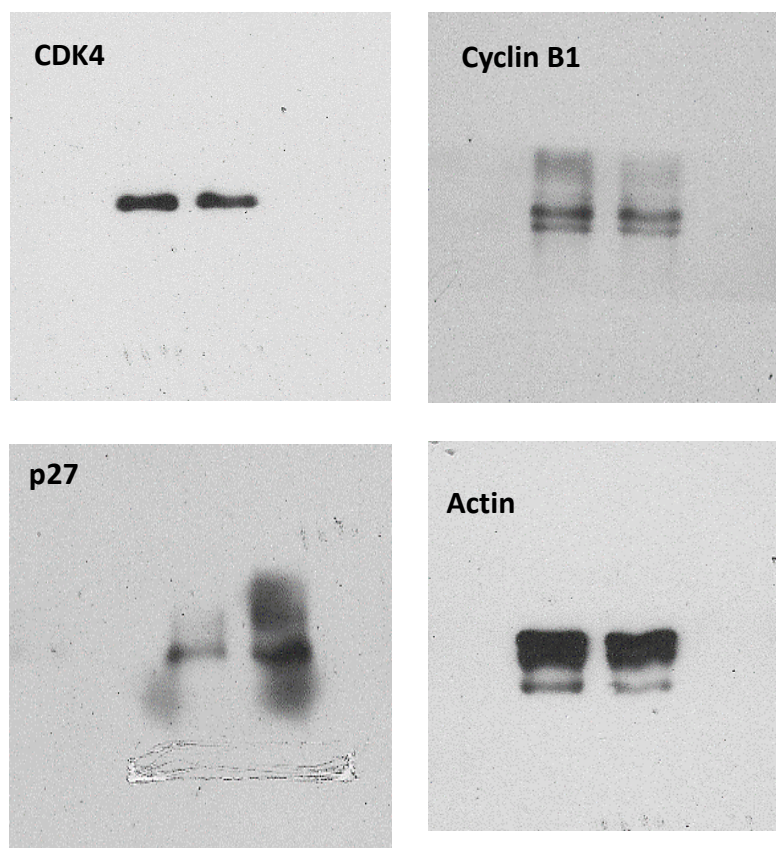

Supplement: Supplementary file 11 — Original western blots [file 41420_2022_1243_MOESM11_ESM.pdf]
