# Supplementary material for: LINC00472 inhibits cell migration by enhancing intercellular adhesion and regulates H3K27ac level via interacting with P300 in renal clear cell carcinoma
Source: Cell Death Discov. 2022 Nov 12;8:454. doi: 10.1038/s41420-022-01243-7 (PMC9653443; doi:10.1038/s41420-022-01243-7)
Supplement: Supplementary file 9 — Supplementary Figure Legends [file 41420_2022_1243_MOESM9_ESM.docx]

**SUPPLEMENTARY FIGURE LEGENDS**

**Figure S1**

**(A)** Verification of constructing scale-free network through WGCNA in normal group. **(B)** WGCNA constructs network based on normal samples and select the soft threshold through scale independence and mean connectivity. **(C)** Verification of constructing scale-free network through WGCNA in cancer group. **(D)** WGCNA constructs network based on cancer samples and select the soft threshold through scale independence and mean connectivity.

**Figure S2**

**(A)** According to the combination of module dissimilarity, 11 sub modules were finally obtained in the normal group. **(B)** The correlation between normal network modules.

**Figure S3**

Relationship between the levels of expression of LINC00472 **(A)**, LINC00152 **(B),** LINC00271 **(C)**, LINC01503 **(D)** and LINC01510 **(E)** and the prognosis of patients (DFS time).

**Figure S4**

**(A)** Cell counts was used to compare the proliferation rate of LINC00472 stable knockdown cell line and control cell line of Caki-1 cells. **(B)** The expression of cell proliferation related proteins in Caki-1 shNC and shLINC00472 cell lines was detected by using western blot. **(C)** Colony formation assay of Caki-1 shLINC00472 cell line and the control cell line. **(D)** Wound healing experiment was used to detect the migration of 769-P shLINC00472 cell line and the control cell line. **(E)** Transwell results showed that the migration of 769-P shLINC00472 cell line was significantly increased comparing with 769-P shNC cell line and **(Left)**. Statistics of migrating cell area percentage **(Right).** In **A** and **E**, data were shown as mean ± SD of three independent experiments (**P<0.01, ***P < 0.001, ns non-significant).

**Figure S5**

**(A)** Western blot was used to detect the levels of cell proliferation related proteins in HK-2 KI-CMV and HK-2 KI-CTR cell line.

**Figure S6**

**(A)** GO enrichment of differentially expressed genes identified in TCGA RCCC. **(B)** KEGG enrichment of differentially expressed genes identified in TCGA RCCC. **(C)** Association between GO enrichment items with significance.

**Figure S7**

**(A)** RT-qPCR was used to detect the mRNA level of these network node molecules in 769-P shNC and shLINC00472 cell line.

**Figure S8**

**(A)** No obvious enrichment peak of H3K4me1 modification was found near the TSS site of ITGB8. **(B)** There was no obvious enrichment peak of H3K9me1 modification near the TSS site of ITGB8. **(C)** Chromatin immunoprecipitation was used to detect the level of H3K4me3 near the ITGB8 TSS site in HK-2-shNC and HK-2-shLINC00472 cell lines. **(D)** Chromatin immunoprecipitation was used to detect the level of H3K27ac near the ITGB8 TSS site in HK-2-shNC and HK-2-shLINC00472 cell lines. In **C** and **D**, data were shown as mean ± SD of three independent experiments (*P<0.05, **P < 0.01, ***P < 0.001, ns non-significant).
