# Supplementary material for: LINC00472 inhibits cell migration by enhancing intercellular adhesion and regulates H3K27ac level via interacting with P300 in renal clear cell carcinoma
Source: Cell Death Discov. 2022 Nov 12;8:454. doi: 10.1038/s41420-022-01243-7 (PMC9653443; doi:10.1038/s41420-022-01243-7)
Supplement: Supplementary file 10 — Supplementary Tables [file 41420_2022_1243_MOESM10_ESM.docx]

**Supplementary Tables**

**Table S1. List of primers used for RNA analyses**

| Gene Name | Primer | Sequence |
| --- | --- | --- |
| LINC00472 | Forward primer | AGCTTTAACCCCAGCCCATT |
|  | Reverse primer | TTTGAGCTGCTCTCCCATCC |
| LINC01626 | Forward primer | CCCCATCATCCCAGTGTGAA |
|  | Reverse primer | GACACATCTCTCTTGGCGCA |
| CDH1 | Forward primer | GCCTCCTGAAAAGAGAGTGGAAG |
|  | Reverse primer | TGGCAGTGTCTCTCCAAATCCG |
| ITGA1 | Forward primer | CCGAAGAGGTACTTGTTGCAGC |
|  | Reverse primer | GGCTTCCGTGAATGCCTCCTTT |
| ITGA6 | Forward primer | CGAAACCAAGGTTCTGAGCCCA |
|  | Reverse primer | CTTGGATCTCCACTGAGGCAGT |
| ITGB8 | Forward primer | CTGTTTGCAGTGGTCGAGGAGT |
|  | Reverse primer | TGCCTGCTTCACACTCTCCATG |
| ITGB2 | Forward primer | AGTCACCTACGACTCCTTCTGC |
|  | Reverse primer | CAAACGACTGCTCCTGGATGCA |
| IGFBP3 | Forward primer | CGCTACAAAGTTGACTACGAGTC |
|  | Reverse primer | CAAACGACTGCTCCTGGATGCA |
| THBS2 | Forward primer | CAGTCTGAGCAAGTGTGACACC |
|  | Reverse primer | TTGCAGAGACGGATGCGTGTGA |
| TNC | Forward primer | ATGTCCTCCTGACAGCCGAGAA |
|  | Reverse primer | AGTCACGGTGAGGTTTTCCAGC |
| WNT5A | Forward primer | TACGAGAGTGCTCGCATCCTCA |
|  | Reverse primer | TGTCTTCAGGCTACATGAGCCG |
| Actin | Forward primer | TGACGTGGACATCCGCAAAG |
|  | Reverse primer | CTGGAAGGTGGACAGCGAGG |

**Table S2. List of primers used for ChIP analyses**

| Name | Primer | Sequence |
| --- | --- | --- |
| ChIP-1 | Forward Primer | CTGCCACTTACAACCCAGCA |
|  | Reverse Primer | GAATGCAGCCACCCTTGAGA |
| ChIP-2 | Forward Primer | ACTCCTCGAATGGGAATCGC |
|  | Reverse Primer | GGTCCTGTCATTACCACGCT |
| ChIP-3 | Forward Primer | TGGTCCAACTCTCCAAAGGC |
|  | Reverse Primer | GCCGCTTTGTTCGGAGATAC |
| ChIP-4 | Forward Primer | AGAGATTGGGGTTTGCTCGC |
|  | Reverse Primer | TGCCATTCCCATCGTTCACA |
| ChIP-5 | Forward Primer | CAAAAGCTCTTTTCTTTGTCCCG |
|  | Reverse Primer | GCGGTGCAGGTAAGCG |
| ChIP-6 | Forward Primer | TAAACAGGGGAGGCCATCCTA |
|  | Reverse Primer | GGTGGTAGAAGTCTCTGGCA |
